# Survival Nomogram for Young Breast Cancer Patients Based on the SEER Database and an External Validation Cohort

**DOI:** 10.1245/s10434-022-11911-8

**Published:** 2022-06-03

**Authors:** Xiao Huang, Zhou Luo, Wei Liang, Guojian Xie, Xusen Lang, Jiaxiang Gou, Chenxiao Liu, Xiangnan Xu, Deyuan Fu

**Affiliations:** 1 Clinical Medical College, Yangzhou University, Yangzhou, Jiangsu Province China; 2 Department of Breast Surgery, Northern Jiangsu People’s Hospital, Clinical Medical College of Yangzhou University, Yangzhou, Jiangsu China; 3 Graduate School, Dalian Medical University, Dalian, China

## Abstract

**Background:**

Young breast cancer (YBC) patients are more prone to lymph node metastasis than other age groups. Our study aimed to investigate the predictive value of lymph node ratio (LNR) in YBC patients and create a nomogram to predict overall survival (OS), thus helping clinical diagnosis and treatment.

**Methods:**

Patients diagnosed with YBC between January 2010 and December 2015 from the Surveillance, Epidemiology, and End Results (SEER) database were enrolled and randomly divided into a training set and an internal validation set with a ratio of 7:3. An independent cohort from our hospital was used for external validation. Univariate and least absolute shrinkage and selection operator (LASSO) regression were used to identify the significant factors associated with prognosis, which were used to create a nomogram for predicting 3- and 5-year OS.

**Results:**

We selected seven survival predictors (tumor grade, T-stage, N-stage, LNR, ER status, PR status, HER2 status) for nomogram construction. The C-indexes in the training set, the internal validation set, and the external validation set were 0.775, 0.778 and 0.817, respectively. The nomogram model was well calibrated, and the time-dependent ROC curves verified the superiority of our model for clinical usefulness. In addition, the nomogram classification could more precisely differentiate risk subgroups and improve the discrimination of YBC prognosis.

**Conclusions:**

LNR is a strong predictor of OS in YBC patients. The novel nomogram based on LNR is a reliable tool to predict survival, which may assist clinicians in identifying high-risk patients and devising individual treatments.

Breast cancer has overtaken lung cancer as the most common type of malignancy globally. In 2020 alone, the number of newly diagnosed breast cancer patients reached 2.3 million, accounting for 11.7% of all cancer cases.^1^ Age is an essential factor for the long-term survival of breast cancer, and young patients often have an inferior prognosis in comparison with other age groups.^2–4^ The ESMO guidelines define young breast cancer (YBC) patients as < 40 years.^5^ YBC patients are relatively rare, making up only approximately 5.6% of all invasive breast cancer patients.^6^ However, numerous studies have revealed that breast cancer in YBC patients is more aggressive (i.e., high tumor grade, common *BRCA1*/*2* mutations, lymph vascular invasion) and is correlated with poorer prognosis.^2–4,7,8^ Given the high level of heterogeneity, the traditional American Joint Committee on Cancer (AJCC) staging system may not predict the survival probability well for YBC patients. Thus, a new prediction tool is needed to assess prognosis accurately for individual planning.

Lymph node ratio (LNR) is defined as the ratio between the number of positive lymph nodes (PLNs) and the total number of resected lymph nodes (RLNs), which has been proposed to improve the prognostic accuracy of lymph node state in various tumors.^9–11^ Likewise, the prognostic value of LNR has also been demonstrated in breast cancer.^12–17^ In several small-sample research studies, LNR even showed better prognostic ability than pathologic nodal stage stratification.^15–17^ Compared with other age groups, YBC patients are more prone to lymph node metastasis.^3,4^ LNR might have particular significance for YBC patients. However, studies related to LNR in YBC patients are rarely reported.

The prognostic role of LNR in YBC has been discussed in a previous report, but the cutoff point of LNR was based on other types of breast cancer instead of YBC.^18^ In our study, LNR was analyzed as a continuous independent variable, and the analysis result was presented through the time-dependent area under the receiver operating characteristic curve (AUC) values. Furthermore, to avoid redundancy or overfitting, LASSO regression was used to screen the most significant factors related to OS for nomogram construction. Compared with the original model, our new nomogram model included fewer variables, creating more convenience for clinical practice. Finally, we internally verified the prognostic performance of the proposed nomogram and carried out an external validation in an independent database.

## Patients and Methods

### Population Selection

The SEER database of the National Cancer Institute is a systematic population-based cancer database that covers about 30% of the population in the USA. In this study, we extracted the data from the SEER 18 registry database using SEER*Stat 8.3.9 software. All the patients we selected had been diagnosed with YBC from 2010 to 2015. The inclusion criteria were as follows: (1) invasive breast cancer patient; (2) female under the age of 40 years; (3) breast cancer as the first primary tumor that was confirmed by histology; (4) underwent surgical treatments. Meanwhile, patients were excluded if: (1) diagnosis with inflammatory breast cancer or Paget's disease; (2) with distant metastasis; (3) bilateral breast cancer; (4) cases without records of follow-up (survival time code of 0 months); (5) missing information on tumor grade, TNM stage, lymph node status, surgery type, ER, PR, and HER2 status. Ultimately, 11,666 eligible patients were included in our study. Referring to previous research, these patients were randomly divided into a training set (*n* = 8166) and an internal validation set (*n* = 3500) in a 7:3 ratio, for the construction and verification of the nomogram,^19,20^ respectively. We consider 7:3 to be an appropriate ratio to apply to this study. Using most of the data to construct the nomogram can ensure the accuracy of the model, while a smaller portion of the data was used for validation to prevent overfitting.

To further validate the proposed nomogram, 351 patients diagnosed with YBC from May 2012 to December 2018 in The Northern Jiangsu People' Hospital (NJPH) were used to form the external validation set. Patients in this validation set were recruited according to the same inclusion and exclusion criteria as the training cohort. The time of last follow-up was November 2021. This study was approved by the institutional review board of NJPH.

### Variable Collection

Several variables were included in the present study: baseline demographics (i.e., age at diagnosis, race, marital status), tumor features (i.e., laterality, histological type, tumor grade, T-stage, N-stage, LNR, AJCC stage, ER status, PR status, HER2 status), therapy information (i.e., surgery, radiation, chemotherapy), and survival variables (i.e., vital status, survival months). We restaged all the included patients according to the eighth pathological edition of the AJCC staging system.^21,22^ The chosen age cutoff value was based on a previously published study.^23^ LNR is defined as the ratio of PLNs/RLNs, and the result is rounded to one decimal place. In our research, the primary outcome was OS, defined as the time interval between date of diagnosis and date of death for all causes.

### Statistical Analysis

Statistical analysis categorical variables are expressed as percentages and continuous variables as the mean ± standard deviation (SD). The time‐dependent AUC curves were used to compare the predictive ability of LNR with the pN-stage. Univariate Cox regression analyses and LASSO regression algorithm were used to screen clinical features significantly related to OS. On the basis of the final results of LASSO Cox regression, a novel nomogram including all the independent prognostic factors was developed to predict 3- and 5-year OS for YBC patients.

To measure the performance of the nomogram, both internal and external validation were used. The C-index and the receiver operating characteristic (ROC) curve were used to evaluate the discrimination of the nomogram. The calibration curves were used to determine the degree of agreement between predicted probabilities and observed outcomes. Both discrimination and calibration were evaluated using bootstrapping with 1000 resamples. The nutrition risk index (NRI) and integrated discrimination improvement (IDI) were used to compare the accuracy capability of the nomogram with that of the traditional AJCC staging system. The clinical usefulness and benefits of the nomogram were estimated by the decision curve analysis (DCA) plots. Furthermore, on the basis of the risk score and X-tile software version 3.6.1 (Yale University, New Haven, CT), all the patients were stratified into low-, intermediate-, and high-risk groups.

In this study, SPSS 25.0 and R software (version 3.6.1) were adopted for all statistical analyses. All *P*-values were two-sided, and *P* < 0.05 was considered statistically significant.

## Results

### Patient Baseline Characteristics

In total, 11,666 eligible patients with YBC were enrolled from the SEER database and randomly assigned to the training set (*n* = 8166) and the internal validation set (*n* = 3500). Meanwhile, 351 cases of patients with YBC from our center were selected and used as the external validation set. The differences between the SEER cohort and the NJPH cohort were mainly in the baseline demographics and the therapy information. For clinicopathologic characteristics, the three groups had only apparent differences in the pathological type (*p* = 0.029). Infiltrating ductal cancer was the most common histopathologic type of YBC (SEER data: 93.6%, NJPH data: 90.9%). High-grade tumors containing poorly or undifferentiated grades were more frequent in YBC patients (SEER data: 56.2%, NJPH data: 55.3%). Moreover, the whole population had a relatively high rate of lymph node metastasis (SEER data: 44.5%, NJPH data: 48.7%). Other clinicopathological characteristics are summarized in Table 1.

**Table 1:** Demographics and clinicopathologic characteristics of the training and validation cohort

Characteristic	Training cohort (*n* = 8,166), *n *(%)	Internal validation cohort (*n* = 3,500), *n* (%)	External validation cohort (*n* = 351), *n* (%)	*P* value
Age (years)				0.669
< 35	3206 (39.3)	1347 (38.5)	141 (40.2)	
≥ 35	4960 (60.7)	2153 (61.5)	210 (59.8)	
Race				< 0.001
White	5860 (71.7)	2512(71.8)	0	
Black	1191 (14.6)	529 (15.1)	0	
Others	1115 (13.7)	459 (13.1)	351	
Marital status				< 0.001
Single	2386 (29.2)	1066 (30.5)	25 (7.1)	
Married	4869 (59.6)	1995 (57.0)	237 (67.5)	
Separated/divorced/widowed	570 (7.0)	267 (7.6)	19 (5.4)	
NOS	341 (4.2)	172 (4.9)	70 (20.0)	
Laterality				0.854
Left	4069 (49.8)	1763 (50.4)	177 (50.4)	
Right	4097 (50.2)	1737 (49.6)	174 (49.6)	
Histology				0.029
IDC	7646 (93.6)	3272 (93.5)	319 (90.9)	
ILC	210 (2.6)	96 (2.7)	7 (2.0)	
Others	310 (3.8)	132 (3.8)	25 (7.1)	
Grade				0.815
I	693 (8.5)	296 (8.5)	24 (6.8)	
II	2866 (35.1)	1255 (35.8)	133 (37.9)	
III/IV	4607 (56.4)	1949 (55.7)	194 (55.3)	
T-stage				0.546
T1	3322 (40.7)	1418 (40.5)	150 (42.7)	
T2	3691 (45.2)	1597 (45.6)	160 (45.7)	
T3	906 (11.1)	396 (11.3)	31 (8.8)	
T4	247 (3.0)	89 (2.6)	10 (2.8)	
N-stage				0.153
N0	4544 (55.6)	1933 (55.2)	180 (51.3)	
N1	2486 (30.4)	1108 (31.7)	109 (31.0)	
N2	731 (9.0)	312 (8.9)	41 (11.7)	
N3	405 (5.0)	147 (4.2)	21 (6.0)	
LNR, median (IQR)	0.00 (0.00-0.20)	0.00 (0.00-0.20)	0.00 (0.00-0.20)	0.552
ER status				0.211
Positive	5934 (72.7)	2537 (72.5)	240 (68.4)	
Negative	2232 (27.3)	963 (27.5)	111 (31.6)	
PR status				0.398
Positive	5218 (63.9)	2223 (63.5)	212 (60.4)	
Negative	2948 (36.1)	1277 (36.5)	139 (39.6)	
HER2 status				0.381
Positive	1961 (24.0)	831 (23.7)	95 (27.1)	
Negative	6205 (76.0)	2669 (76.3)	256 (72.9)	
Surgery				0.004
BCS	2415 (29.6)	1076 (30.7)	78 (22.2)	
Mastectomy	5751 (70.4)	2424 (69.3)	273 (77.8)	
Radiotherapy				< 0.001
No/unknown	4040 (49.5)	1721 (49.2)		
Yes	4126 (50.5)	1779 (50.8)		
Chemotherapy				< 0.001
No/unknown	1605 (19.7)	686 (19.6)	16 (4.6)	
Yes	6561 (80.3)	2814 (80.4)	335 (95.4)	

Data expressed as *n* (%) unless otherwise specified

*IQR* interquartile range, *IDC* invasive ductal carcinoma, *ILC* invasive lobular carcinoma, *BCS* breast-conserving surgery

### Time‐Dependent AUC Curves for LNR and pN-Stage

**Fig. 1:**
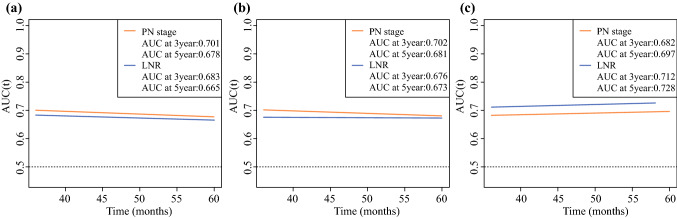
Time-dependent AUC values of pN stage and LNR for the prediction of OS in the training set **(a)**, the internal validation set **(b)** and the external validation set **(c)**.

On the basis of the cumulative sensitivity and dynamic specificity, the time-dependent AUC curves were plotted for OS status. Figure 1 illustrates the changes over time for AUC. In the patients diagnosed with YBC from the SEER database, the AUCs of OS were slightly better for the pN classification system than for LNR. However, as in other studies, LNR showed better prognostic power than the pN-stage in the patients from our center.^15–17^

### Feature Selection and Nomogram Construction

**Fig. 2:**
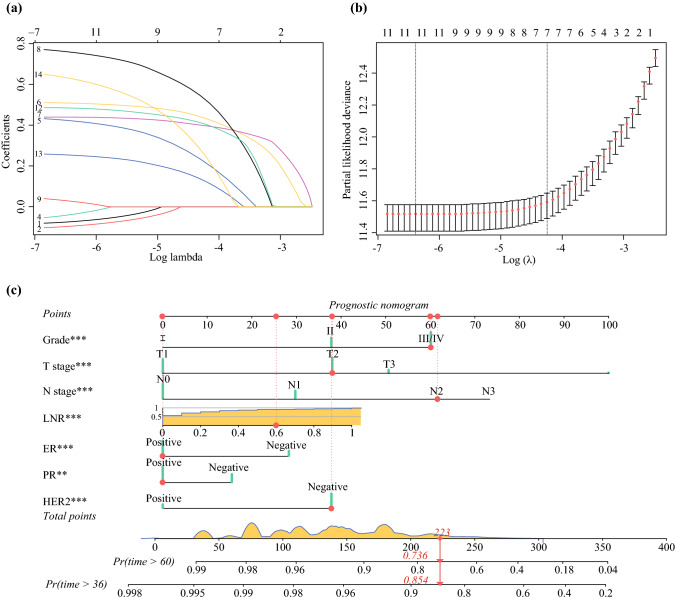
LASSO Cox regression model construction. **a** LASSO coefficients of fourteen features; **b** Selection of tuning parameter (λ) for the LASSO model. **c** Nomogram for predicting 3- and 5-year OS in YBC patients.

A total of 15 clinical parameters were included in the training set. In the univariate Cox regression analysis, only laterality was not associated with OS (*P* = 0.780). The variables that reached the prognostic significance in the univariate analysis were included in the LASSO regression. Among them, seven factors (i.e., tumor grade, T-stage, N-stage, LNR, ER, PR, and HER2 status) with nonzero coefficients were ultimately considered as the statistically significant factors related to OS (Fig. 2a, b). On the basis of these seven significant variables, a nomogram for predicting 3- and 5-year OS of YBC patients was developed (Fig. 2c). To use the nomogram, each level of these variables was assigned a specific point on the scale. By summing the points from each variable, a total point was obtained for the individual patients. We can then predict 3- and 5-year OS probability by projecting the total points to the total score scale of the nomogram. For instance, for a young patient (< 40 years old) diagnosed with a grade III, T2N2, LNR 0.6, ER positive, PR positive and HER2 negative breast cancer, the total point for all variables was 223, which corresponded to 3- and 5- year OS rates of about 85.4% and 73.6%, respectively.

### Performance and Validation of the Nomogram

**Fig. 3:**
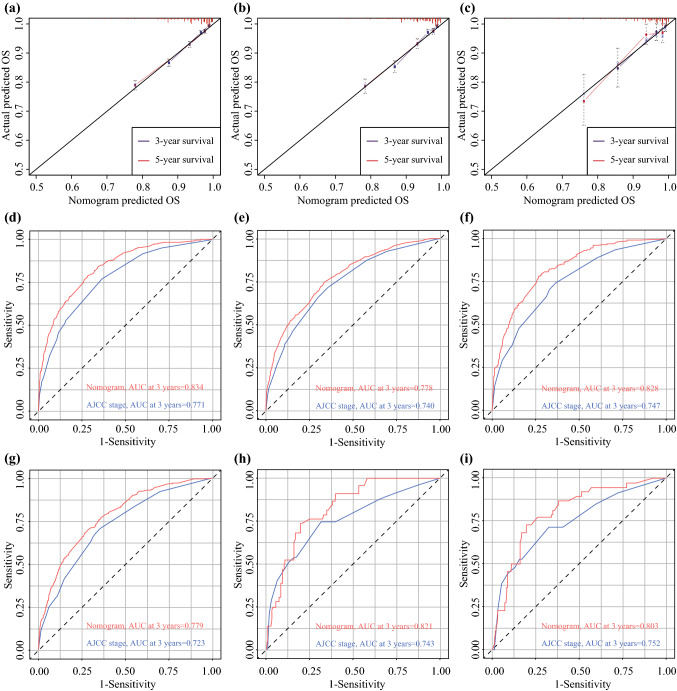
The calibration curves to predict 3- and 5-year OS in the training set (**a**), the internal validation set (**b**) and the external validation set (**c**). Time-dependent ROC curves comparing the use of the nomogram and AJCC TNM staging system to predict the 3- and 5- year OS for YBC patients in the training set (**d,e**), the internal validation set (**f,g**) and the external validation set (**h,i**), respectively.

The calibration curves of the nomogram showed high uniformity between the predicted and actual probabilities of OS in the training set (Fig. 3a), the internal validation set (Figure 3b), and the external validation set (Fig. 3c). The C-indexes values based on the nomogram (training set, 0.775; internal validation set, 0.778; external validation set, 0.817) were higher than those based on the AJCC stage (training set, 0.735; internal validation set, 0.719; external validation set, 0.751). Meanwhile, time-dependent ROC curves at 3- and 5-years showed that the nomogram performed better in predicting the prognosis of OS than the traditional AJCC staging system, respectively, in the training set (Fig. 3d, e), the internal validation set (Fig. 3f, g) and the external validation set (Fig. 3h, i).

**Fig. 4:**
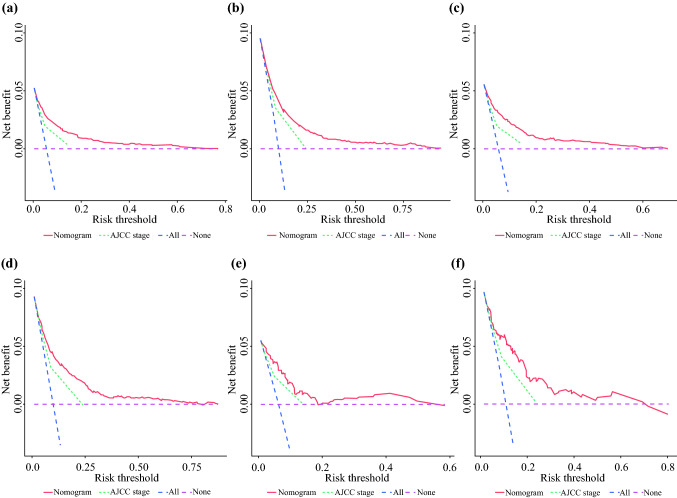
DCA curves of the nomogram and AJCC TNM staging system for predicting 3- and 5-year OS in the training set **(a,b)**, the internal validation set **(c,d)** and the external validation set **(e,f)**.

DCA was performed to compare the clinical applicability of the nomogram with that of the traditional AJCC staging system. As shown in Fig. 4, DCA curves demonstrated that nomogram could better predict the 3‐ and 5‐year OS, as it added more net clinical benefits compared with the AJCC stage model in all three cohorts.

Subsequently, NRI and the IDI were further used to compare the accuracy between the nomogram and the traditional AJCC staging system. In the training set, the NRI for 3- and 5-year OS were 0.257 (95% CI 0.208–0.345) and 0.190 (95% CI 0.124–0.237), and the IDI for 3- and 5-year OS were 0.086 (95% CI 0.068–0.109, *P* < 0.001) and 0.085 (95% CI 0.070–0.105, *P* < 0.001). These results were validated in the internal validation set and the external validation set (Table 2), suggesting that the nomogram predicted OS with greater accuracy than the traditional AJCC staging system.

**Table 2:** NRI and IDI of the nomogram and the traditional AJCC staging system in OS prediction for YBC patients

		NRI		IDI	
		3-Year	5-Year	3-Year	5-Year
*Training cohort (n = 8166)*					
Estimate		0.257	0.190	0.086	0.085
95% CI		0.208–0.345	0.124–0.237	0.068–0.109	0.070–0.105
*P* value				< 0.001	< 0.001
*Internal validation cohort (n = 3500)*					
Estimate		0.257	0.220	0.092	0.090
95% CI		0.192–0.342	0.121–0.306	0.067–0.131	0.066–0.123
*P* value				< 0.001	< 0.001
*External validation cohort (n = 351)*					
Estimate		0.140	0.131	0.079	0.105
95% CI		0.008–0.453	0.058–0.533	0.026–0.211	0.047–0.248
*P* value				< 0.001	< 0.001

### Risk Stratification Ability Assessment of the Nomogram

**Fig. 5:**
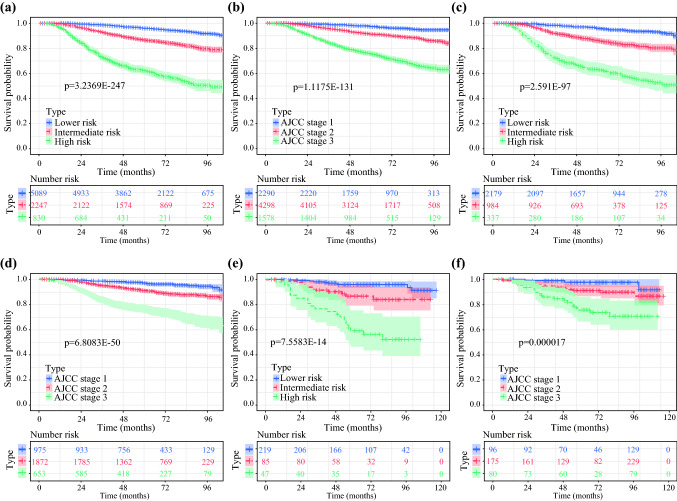
Kaplan-Meier curves of OS for risk stratification and defferent AJCC stages in the training set **(a,b)**, the internal validation set **(c,b)** and the external validation set **(e,f)**.

Finally, we calculated the risk score for every patient through the nomogram and made a risk stratification. On the basis of the cutoff values made by X-tile software, all patients with YBC were divided into three risk subgroups: low risk (points ≤ 155), intermediate risk (155 < points ≤ 214), and high risk (points > 214).^24^ The Kaplan–Meier survival curves revealed obvious discrimination among different risk subgroups, whereas the traditional AJCC staging system had limited capability to identify high-risk patients in all three cohorts (Fig. 5).

## Discussion

The incidence of breast cancer in young women is relatively low.^6^ However, compared with older patients, young breast cancer patients typically have poor prognosis.^2–4^ In this study, we explored the clinicopathological features and prognostic factors of YBC patients using the SEER database and the independent data from our center. In addition, seven significant factors associated with prognosis were identified through LASSO regression and were used to construct a new nomogram to predict survival in YBC patients. Finally, our study demonstrated that the nomogram outperformed the AJCC staging system in predicting 3- and 5-year OS of these individuals on both internal and external validation cohorts.

Lymph node status in breast cancer is widely accepted as an important predictor for patient prognosis.^25,26^ Traditionally, the number of PLNs was deemed as the most significant prognostic factor in breast cancer, and formed the foundation of the pN category of the AJCC staging system.^21^ However, many factors may affect the number of examined lymph nodes, such as varied levels of surgical expertise and different handling of the surgical specimen by the pathologist. The tumor stage could be underestimated when the number of resected and assessed lymph nodes is insufficient, which might lead to inadequate treatment and incorrect prognostic judgment.^27^ To tackle this problem, LNR has been introduced to assess the prognosis in breast cancer.^12–17^ Many studies have shown that treating LNR as a categorical variable will weaken the prognostic power, and it is better to assess LNR as a continuous variable to reveal its true performance.^28,29^ We agreed with this view and analyzed LNR as a continuous variable. In our study, LNR exhibited excellent predictive capability in YBC patients, especially in the external validation set. Notably, LNR revealed a better survival predictive ability than the pN-stage in the data obtained from our center, which was in line with the results of previous studies.^15–17^ We consider that LNR might perform better than the pN-stage for predicting prognosis in the single-institution study with a small sample size. However, more research is required to confirm this conjecture.

In 2020, through univariate and multivariate Cox analyses, Yi and colleagues developed a nomogram that included 13 predictors to predict the survival probability for YBC patients.^18^ However, we considered that too many predictors are unnecessary for clinical application because the inclusion of variables that are not significantly related to the outcome contributed little to the improvement of the model. Compared with the traditional multivariable regression, LASSO regression was considered as a better method to select variables since it can minimize overfitting and reduce the complexity of the model by using a loss function or penalty term that is added to the objective function.^30,31^ Through the LASSO regression algorithm, only seven variables (i.e., tumor grade, T-stage, N-stage, LNR, ER, PR, and HER2 status) were identified as the independent factor associated with OS in our study. On the basis of these variables, we constructed a more parsimonious nomogram, which greatly ameliorated the clinical applicability in clinical scenarios. In addition, the novel nomogram with fewer variables also performed very well in both internal and external verification.

Among the seven parameters included in our nomogram, the T-stage made the most significant contribution to OS. LNR and the pN-stage cooperated with each other to reflect the status of lymph nodes so as to better predict the prognosis of patients. In addition, tumor grade, ER, PR, and HER2 status were identified as prognostic factors of YBC, consistent with the results of previous studies.^4,18^ Nonsignificant factors, such as race and marital status, were excluded in the nomogram, which helped to save time and energy for the physician in collecting unnecessary information. In addition, adjuvant therapies, including radiotherapy and chemotherapy, were not considered as independent factors in LASSO regression, possibly because they were generally associated with poor tumor features rather than treatment failure.

The nomogram that we developed exhibited a significantly stronger capability in risk stratification for YBC patients than the current AJCC eighth edition, which can be used for patient consultation on survival information, guiding clinical decision making and treatment allocation. Patients defined as high risk through the nomogram are expected to have a dismal prognosis, so we recommend that these patients should receive additional treatment and intensive follow-ups. Furthermore, in current clinical practice, multigene tests, such as the 21-gene recurrence score (21-RS) and the 70-gene signature (70-GS), are currently being used to predict recurrence and survival, and identify candidates for adjuvant chemotherapy among young women with early-stage hormone receptor-positive and HER2-negative breast cancer.^32,33^ We suggest that the combination of the nomogram and genomics might better guide clinical decision-making for this subset of patients.

There exist several limitations in the present study. Firstly, this is a retrospective study based on the SEER database and NJPH database; as such, selection bias is unavoidable. Also, certain important information, such as Ki-67 index, *BRCA1*- and *BRCA2*-related mutation and endocrine therapy, is unavailable in the SEER database, the absence of which might reduce the predictive power of individual prognosis among YBC patients. Lastly, young age is associated with higher risk of recurrence.^34^ Unfortunately, the SEER database does not provide information about disease recurrence. Thus, the recurrence risk of YBC patients could not be assessed in our study.

## Conclusions

For YBC patients, LNR can be regarded as a powerful prognostic factor. On the basis of LNR, we constructed a nomogram to provide a convenient and reliable tool for predicting OS in YBC patients, which would contribute to identifying high-risk patients for physicians.
